# Contraceptive Use, Access to Methods, and Program Efforts in Urban Areas

**DOI:** 10.3389/fgwh.2021.636581

**Published:** 2021-09-23

**Authors:** John A. Ross

**Affiliations:** Independent Demographic Consultant, New Paltz, NY, United States

**Keywords:** contraception, access, program effort, regions, equity

## Abstract

This article uses two large sets of internationally comparable national surveys to analyze urban patterns of contraceptive use, access to methods, and fertility. Urban areas show higher use of contraception and lower fertility rates than rural areas, with substantial differences in the method mix. Urban women tend more toward the long-term methods of the intrauterine device (IUD), implant, and sterilization and less toward short-term, resupply methods. The overall use increases with education and with higher wealth quintiles. By regions, contraceptive use is unbalanced between urban and rural sectors especially in sub-Saharan Africa, where the overall levels of use are lowest. Overall, the urban fertility rate is only 70% of the rural rate. Across countries, the fertility rate correlates negatively with contraceptive use. National family planning programs tend to raise contraceptive use and to improve access to the methods. About half of the unmarried, sexually active women use contraception, with great diversity in which method is preferred. This holds for all married women as well: regions and countries show quite different patterns of use. Therefore, planners and donors should focus on the circumstances in individual countries.

## Introduction

The conditions under which most people live have been transformed over the last seven decades by what may be termed the “urban revolution.” Since the end of World War II, the proportion of people dwelling in cities has grown from less than a third (30%) in 1950 to over half (56%) in 2020, and it is projected to exceed two-thirds (68%) by 2050. In terms of numbers, the urban population grew from 750 million in 1950 to 4.4 billion in 2020, and it is projected to total 6.7 billion by 2050, nearly nine times higher than in 1950. Meanwhile, the total rural population, which grew from 1.8 billion in 1950 to 3.4 billion in 2020, is expected to decline by about 10%, from 3.4 billion in 2020 to 3.1 billion by 2050 (1)^1^.

The urban sector is projected to grow in many different ways across countries and regions. In Latin America, over the 30 years from 2020 to 2050, the urban population is projected to increase from 539 million to 685 million, an increment of 146 million for a 27% increase. Sub-Saharan Africa, starting from a smaller 2020 total of 459 million, will grow to 1.26 billion, an addition of 800 million, up by 174% (1).

Uneven growth is illustrated by the different paths expected in China and India; in 2020 they accounted for 40% of residents in developing regions. The United Nations (UN) projections to 2050 show urban populations in China increasing between 2020 and 2050 by 217 million, up by a fourth (25%; 875 million to 1.092 billion). India will exceed this by a large margin, increasing by 394 million, up by four-fifths (81%) in the same period (483 million to 876 million). Together, China and India alone will account for 28% of all growth by 2050 in the developing regions. The next 10 countries^2^ (by the size of their 2020 urban populations) will account for another 18%. To sum up, 46% of all growth will occur in only 12 countries.

The massive shift into the cities raises the demands for public services, housing, education, and jobs in a frequently losing battle with the ever-increasing numbers. In the case of reproductive health and contraceptive protection of women, medical infrastructures must grow, as well as the capacities of the private sector to address the needs for both short-term and long-term methods.

The literature is unfortunately scanty on urban–rural differences in contraceptive use for large sets of low- and middle-income countries. Many studies on local and individual countries exist, but they are highly variable in character and no attempt is made to review them here. Instead, the available cross-country compilations that compare urban and rural sectors for variables of interest are discussed.

Two early UN publications compiled numerous national surveys and discussed the urban–rural differentials in contraceptive use. The first (2) used 20 surveys from the World Fertility Surveys^3^ (WFS), a series running from 1974 to 1987 that ultimately included 42 countries and led to the Demographic and Health Survey (DHS) program. The 1981 study found considerably higher use rates among urban than among rural women for nearly all countries, though somewhat smaller differences after controls for education. The perceived availability of services appeared to matter since they paralleled the differentials in use in countries with evidence on both.

The second UN publication (3) used WFS surveys in 30 countries and found similar urban–rural differentials in contraceptive use, but it expanded the work to examine use according to the method. In most of the countries, the use of the long-term methods of sterilization and the intrauterine device (IUD) differed little between sectors, but in cities, short-term supply methods were favored, as opposed to the traditional methods in rural areas.

In 2011, the UN published a Wall Chart covering all countries, with 17 indicators across a range of topics for the urban sector (4). These indicators included urban population sizes and growth rates, the shares of the urban population dwelling in the largest cities, the percentages living in slums, and the percentages using improved sanitation facilities and improved drinking water sources. They also included carbon dioxide emissions and certain development measures such as GDP per capita, the percentage working in industry and services, and the energy consumption per capita.

Also, in 2011, the UN published an update of its series that provided estimates and projections of the numbers of urban residents in all countries, by individual year, from 1950 through 2050. For the first time, this update incorporated geographic coordinates for large cities (633 cities with over 750,000 inhabitants). This permitted linkages to data on various environmental characteristics such as nearness to coasts, earthquake faults, and climate zones.

The most recent report in the series, for 2018, is available at https://population.un.org/wup/. In addition, the UN maintains an online database that permits access to each country, with options for data tables, figures, and maps.

In 2015, the Population Reference Bureau (5) published a Wall Chart with separate urban and rural values on 16 indicators for low- and middle-income countries. These indicators are categorized under the five headings of demography, socioeconomic variables, maternal and child health, family planning, and drinking water and sanitation. Important findings included the earlier start of childbearing and less antenatal care in rural areas, as well as less skilled attendance at delivery together with higher infant mortality rates. In addition, more children below age 5 were underweight. Rural areas suffered from less access to improved drinking water and less access to improved sanitation facilities.

As part of its long-running annual series of Wall Charts, the PRB has most recently included the percentage of the population living in cities and the percentage living in cities of 1 million or more for each country (6).

In summary, the available cross-national literature provides numbers and projections for the rural and urban populations, in all countries, to 2050. Much is known about the use of contraception in whole populations, but the picture just for the urban sector is often not available, and no common source has been found that breaks down the urban contraceptive use by the personal characteristics of users.

The present study uses the DHS series to compare the rural and urban sectors for contraceptive use by method, by six geographic regions, covering most of the developing world. Urban–rural ratios for contraceptive use are given for each country. The patterns of use according to four personal characteristics are provided, along with the method preferences of unmarried sexually active women. The association of urban fertility levels with contraceptive use is discussed. Finally, the efforts of national family planning programs to extend contraceptive use are presented, with estimates of the proportions of populations with access to each major contraceptive method.

## Methods and Data Sources

The analyses in this article draw upon a set of 87 countries included in the DHS series, a long-running program that collaborates with agencies in many countries, primarily in the developing world, to assess a range of measures for reproductive health, contraceptive use, fertility, and related items. The DHS series uses nationally representative surveys with large sample sizes (usually between 5,000 and 30,000 households). These focus on women aged 15–49, and in some countries, men aged 15–54 or 59 are interviewed in a subsample. Most of the countries interview women of all marital statuses; all countries cover at least the married in-union women, and they are the focus here to include more countries for analysis. Some countries also interview sexually active unmarried women below age 30 or 25, and this data set is used in one section below. (For further information on the DHS series, see https://dhsprogram.com/). Alternative sources such as the MICS, PAPCHILD, and RHS series^4^ do not contain urban–rural breakdowns for personal characteristics and other items needed for the analyses in this article, focusing especially on the urban sector.

To examine the use of contraception, the main eight contraceptive methods are studied: the “modern methods” of male and female sterilization, IUD, implant, pill, injectable, condom, and the traditional methods (rhythm and withdrawal). Other methods with little use in most of the countries are omitted. Personal characteristics of contraceptive users are included by age, family size, education, and wealth quintiles. Of the survey dates, one-sixth are between 1985 and 1999, another-sixth are from 2000 to 2009, and two-thirds are from 2010 to 2018. Surveys come from 7 regions: 15 from Asia, 5 from the Central Asia Republics, 3 from Europe, 15 from Latin America, 8 from the Middle East/North Africa, 19 from the East and Southern countries of sub-Saharan Africa, and 22 from the West and Central countries of sub-Saharan Africa. Only 55 countries have data on the unmarried, sexually active group of women, and only 65 have information on contraceptive use by the personal characteristics of age, the number of living children, education, and wealth quintiles. Surveys done prior to 2000 are omitted in the figures and tables but are included in the Appendices. Trends as such are not analyzed; the focus is on the most recent picture as approximated by the latest available surveys in the DHS series, which uniquely affords the urban–rural comparison not available in other large-scale data sets.

The urban–rural breakdown follows the definition in each country; this varies, as many go according to the population size of each locality, while others reflect the levels in the administrative structure. The compilations by the UN and other agencies, as well as the DHS series, necessarily use the definition available in the data set of each country.

An additional source of data, besides the DHS series, is employed to study the relation of contraceptive use to the strength of national family planning programs. This data set comes from a 2014 study of 90 countries that rated the strength of program efforts under the components of policies, services, evaluation, and access to methods.

## Results

### Contraceptive Prevalence by Method and Residence

**Figure 1:**
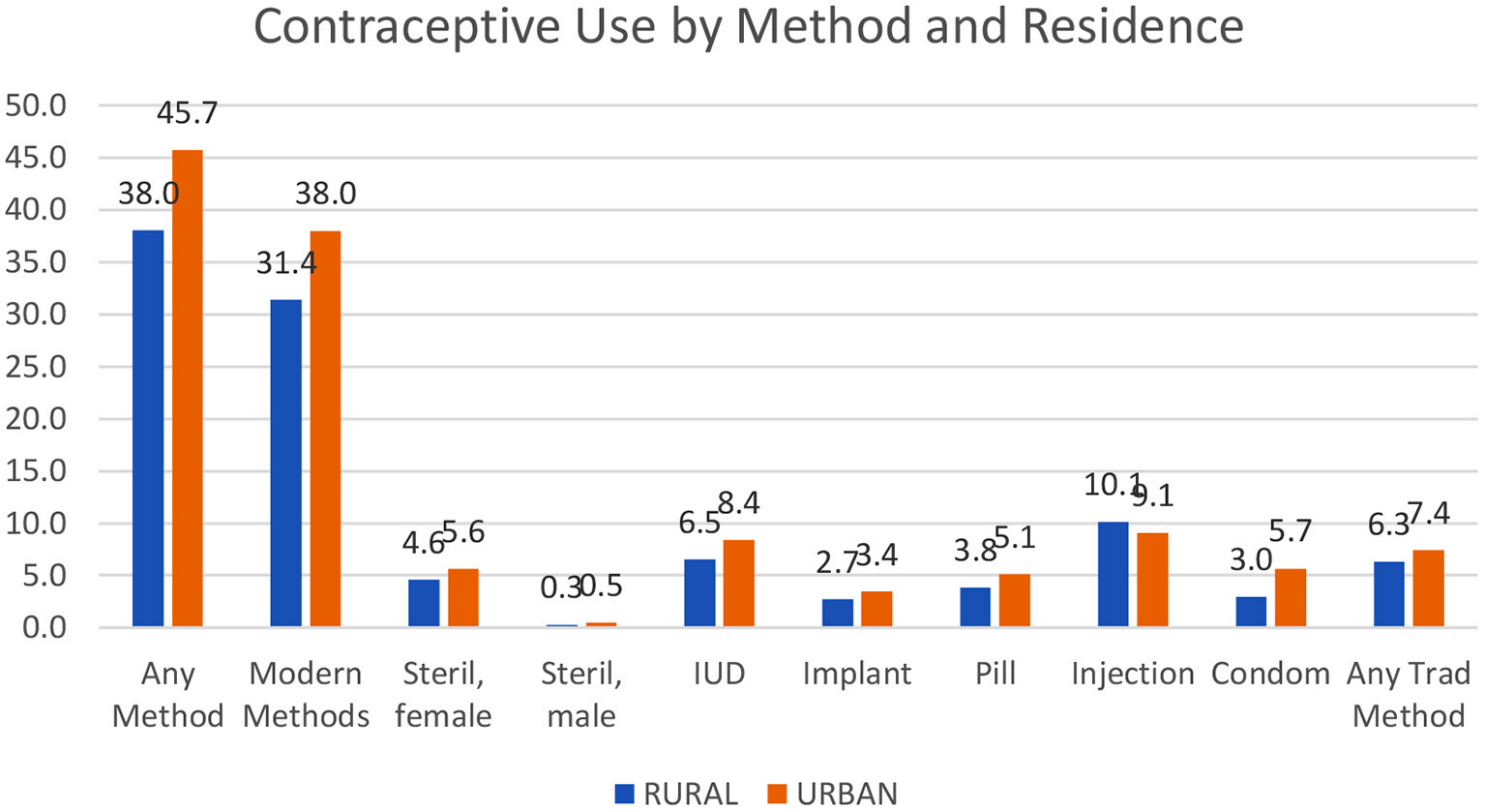
Contraceptive use by method and residence.

Contraception is used more in urban than in rural areas, quite consistently and by a substantial margin. For “any method” at left in Figure 1, the gap is 7.7 percentage points (45.7 vs. 38.0%) for any method and 6.6 percentage points for modern methods (38.0 vs. 31.4%).

In four of five countries, contraception is used more in urban than in rural areas. The actual levels of urban use for any method vary greatly. The lows occur especially in the West and Central parts of sub-Saharan Africa at a mere 11.2% in Chad, 13.0% in the Gambia, and 16.0% in Guinea. The highest levels are in Asia with Vietnam at 79.1% and in Latin America with Brazil (78.7%), Colombia (81.5%), and Peru (75.8%) (Appendix 1).

Figure 1 and the following figures use only the surveys conducted in 2000 or later.

The urban advantage is true for each of the eight contraceptive methods except the injectable; even the traditional methods (rhythm and withdrawal) are used more in the cities. The urban advantage is greatest for the condom, by 2.7%, and the IUD, by 1.9%. Appendix 1 includes contraceptive use by the method and residence for all countries.

(The averages in Figure 1 are unweighted, giving each country the same importance, so a small country counts as much as a large one. With population weights, the pattern is the same, but the levels would differ. The largest countries then dominate. For example, the top four countries in Asia, namely, India, Pakistan, Bangladesh, and Indonesia contain two-thirds of all Asia outside of China).

### Ratios of Contraceptive Use by Residence

**Figure 2:**
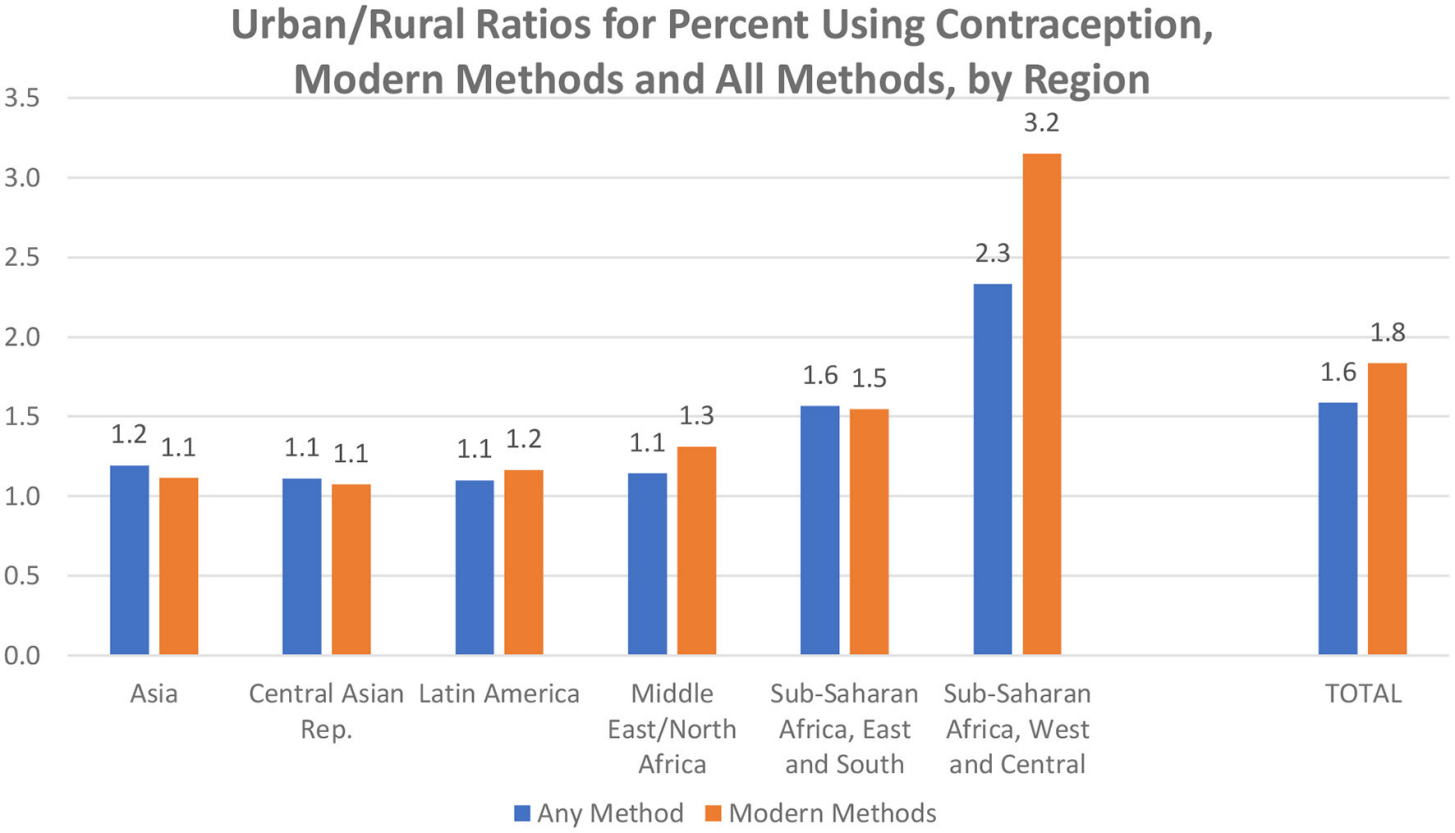
Urban/rural ratios for percent using contraception, modern methods and all methods, by region.

The urban excess in contraceptive use varies considerably by region. The urban/rural ratios (Figure 2) are the highest in sub-Saharan Africa and especially in its West and Central region, where all use is at lower levels and the infrastructures are less advanced. For all methods, the ratio there averages 2.3 and for modern methods, the ratio averages 3.2.

Overall (total bars), the urban advantage is greater for modern methods than for all methods. A contributing factor may be the easier logistics for resupply methods and access to clinical methods in the cities than in the outlying areas. Appendix 2 provides the urban-rural ratios by method, for all regions and countries.

### Ratios by Country

**Figure 3:**
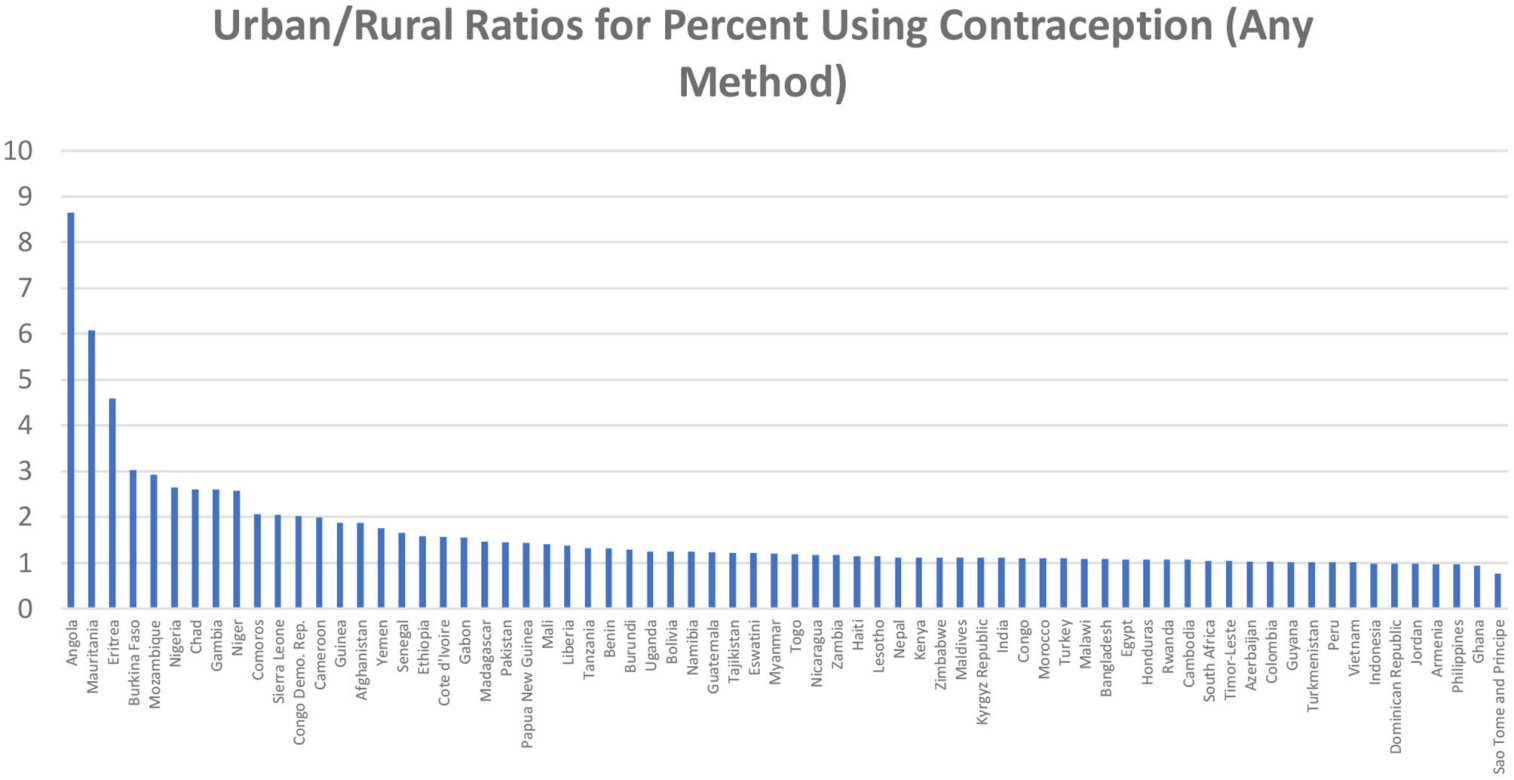
Urban/rural ratios for percent using contraception (any method).

Behind the regional averages lie the considerable country differences. Figure 3 based on Appendix 2 sequences countries by the ratio size (median 1.2).

Greater use in the cities is common, by 50% or more in many countries (ratio of 1.5 or higher). The ratios reach 2 or 3 or more in 13 countries; three outliers exceed 4. All are in sub-Saharan Africa, including the two large countries of the D.R. Congo and Nigeria. The original levels of use there tend to be lower for both urban and rural sectors, and the rural sectors suffer from poorer health infrastructures vital for contraceptive services. Ratios closer to equality occur where contraceptive prevalence is relatively high, as in Indonesia, Peru, Colombia, and Vietnam.

### Use by Personal Characteristics

**Figure 4:**
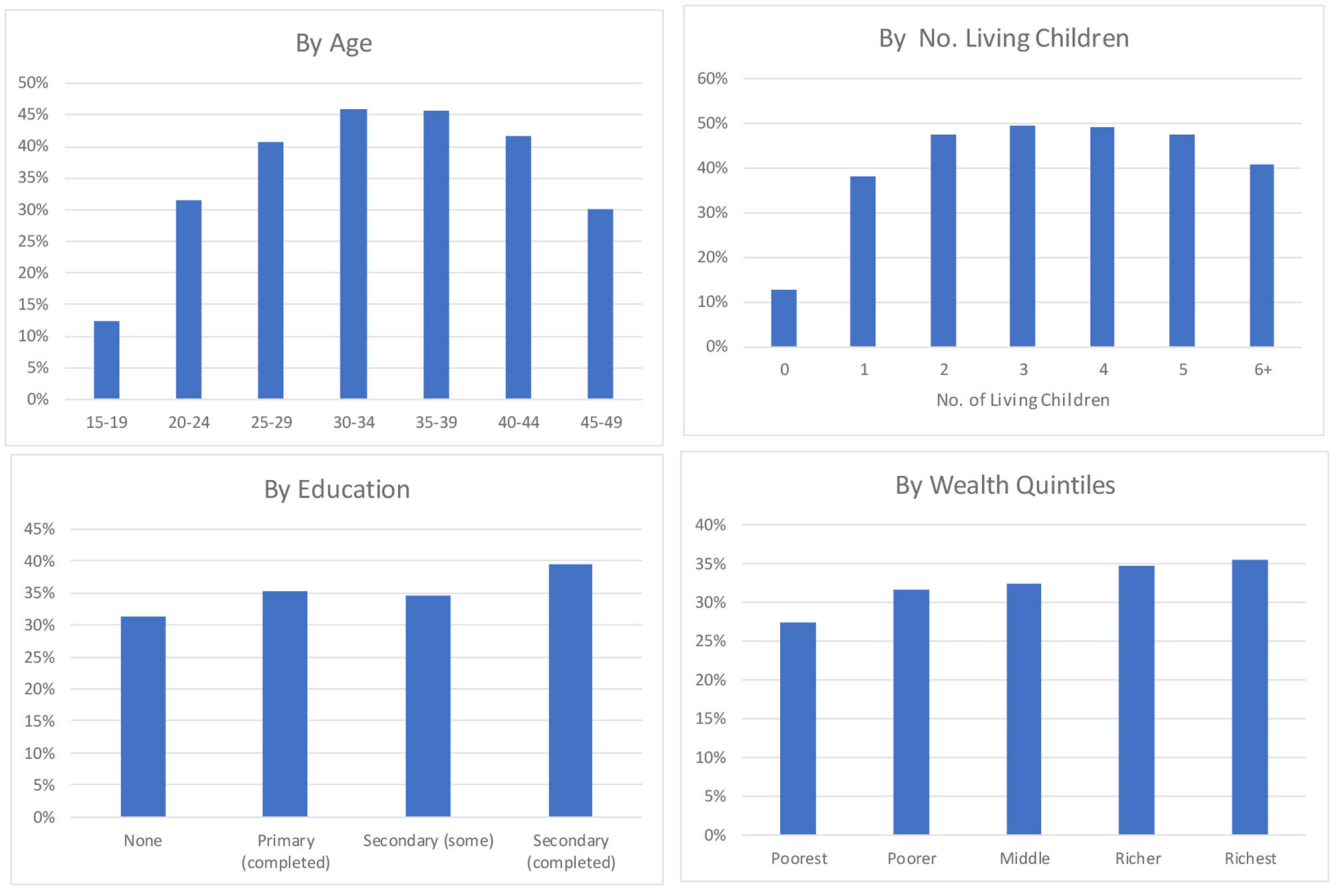
Differentials in the percentage using any method of contraception by four personal characteristics, urban sector.

It is of interest to know how contraceptive use varies by the personal characteristics of age, family size, education, and wealth (Figure 4). As above, these show averages across the 87 countries, just for the urban sector and just for married and in-union women.

The age pattern is as expected, showing the common bell-shaped pattern, with the greatest use centering on women in their 30's, where many wish to cease childbearing and others are spacing to postpone the next birth.

By the number of children, the pattern of use is similar to that for age, except for more uniformity across the groups. The level of 45–50% obtains for all four middle groups. Moreover, the use is sustained in the highest group at over 40%, compared to that at about 30% for the highest age. Note the interaction between contraceptive use and the number of children: for some women, a larger family motivates them to use a method, but for others, the failure to use something causes the larger family.

Women of higher education use contraception by a clear margin. Differences are small between those of “some primary” and those of “some secondary” levels, and the least use occurs among those with no education. Overall, 35% or about a third are using some method.

Contraceptive use according to wealth quintiles^5^ is greatest among the richest group of women, but it is nearly as great in the next level down. It then drops in the next two groups and is least in the bottom group. There is a nine-point difference overall between 27 and 36%. Thus, while the overall use is greater in the cities, there is the expected differential in the use by relative wealth.

**Figure 5:**
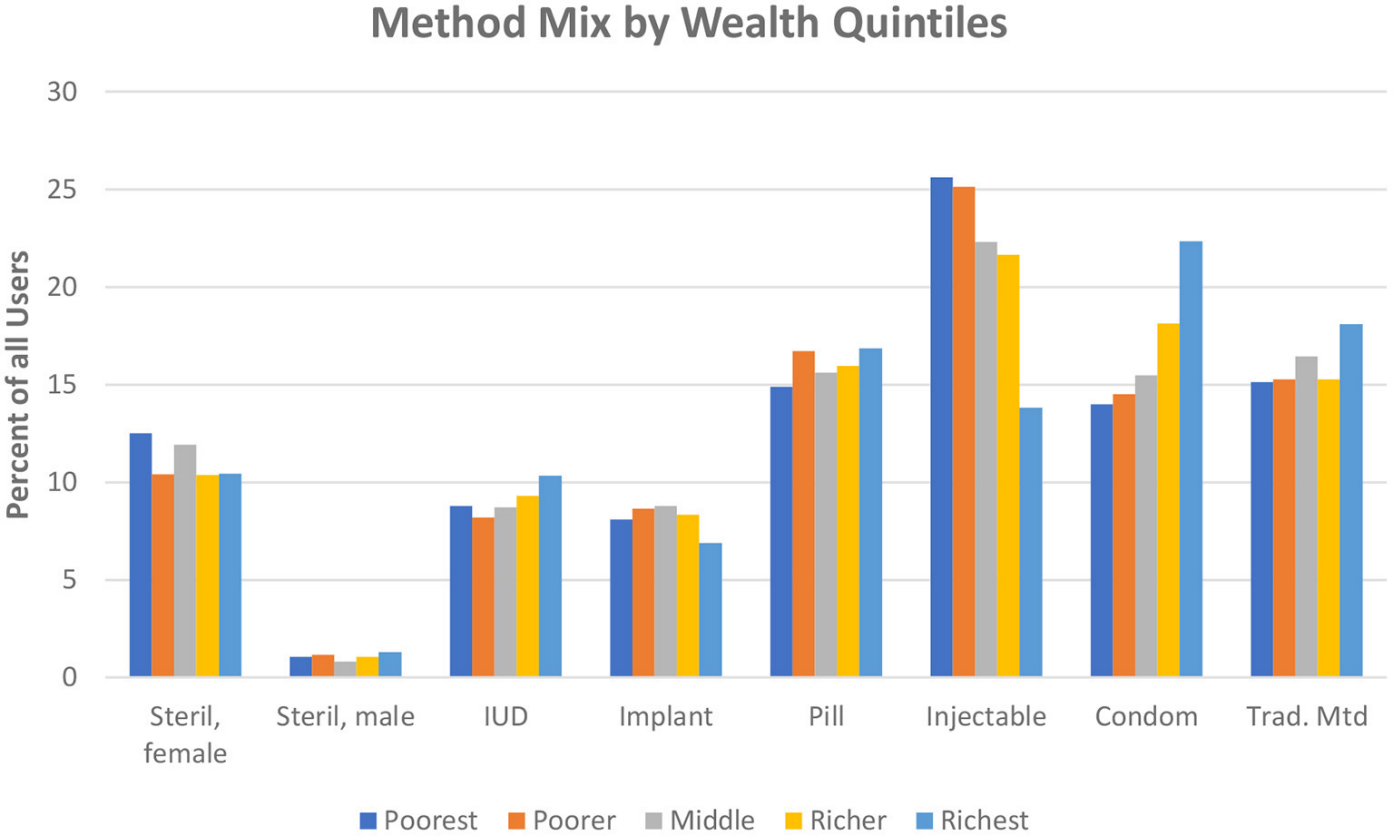
Method mix by wealth quintiles.

The quintile comparison is extended to show differences by the method mix (Figure 5) since public action programs must be shaped partly by the characteristics of the poorest women, who live disproportionately in slum dwellings and need to be contacted in special ways by staff and the media (In the figure, tracing each quintile across the bars, all methods add to 100%).

Two notable results are the preferences by the poorest quintiles for the injectable and their aversion to the condom. They have a slight aversion to the IUD and the pill. The bars for the richest women are highest for the condom, next with small differences among the traditional methods, pill, and injectable. Levels for the richest quintile are lower for the implant, IUD, and female sterilization and are negligible for male sterilization. This is a general pattern: four methods at the left are least favored, while four methods to the right are most favored.

### Contraception and Fertility

The method mix as discussed above affects the fertility rate since certain methods have higher failure and discontinuation rates. However, the total prevalence of use matters, and overall, fertility rates are lower where more women use contraception, documented below for the urban sector.

**Figure 6:**
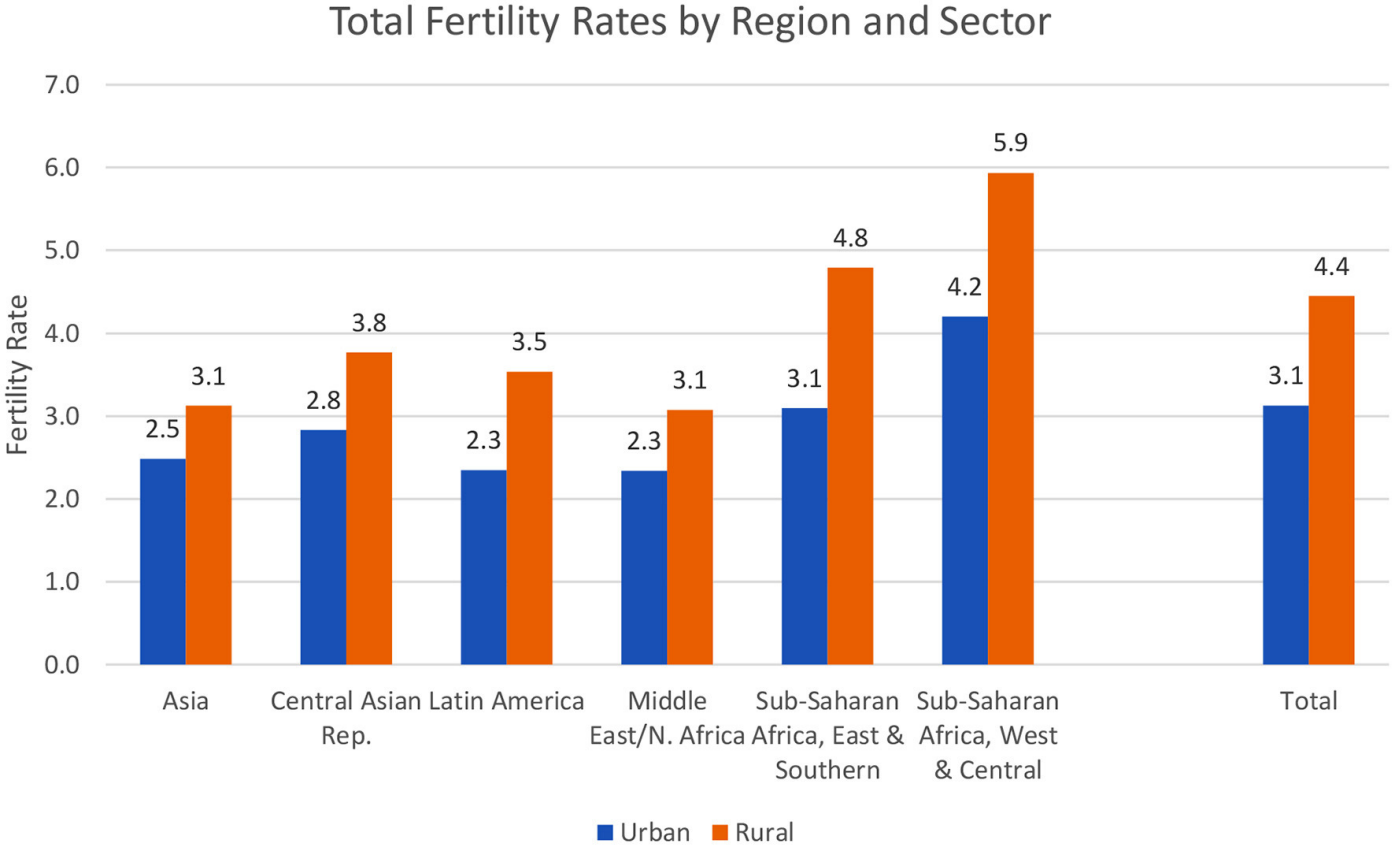
Total fertility rates by region and sector.

Rural fertility is higher, by large margins, according to the total fertility rate (TFR), the average number of lifetime births expected from the current age patterns. The general pattern (Figure 6) is true within every geographic region. Overall, the average TFR is 3.1 for urban women vs. 4.4 for rural women, higher by nearly a half (ratio of 1.42).

Rural fertility is far higher than urban fertility in some of the same countries that show a similar imbalance in contraceptive use. The TFR ratio is above 4 in 11 sub-Saharan African countries including Nigeria, Niger, DR Congo, and Mauritania. It is above 3 in Tanzania, Rwanda, and Uganda, among others. The lowest ratios are in countries with low TFR levels; they fall at 2 or below in Bangladesh, Nepal, and Colombia, as well as in the former USSR republics of Armenia, Moldova, and Ukraine.

**Figure 7:**
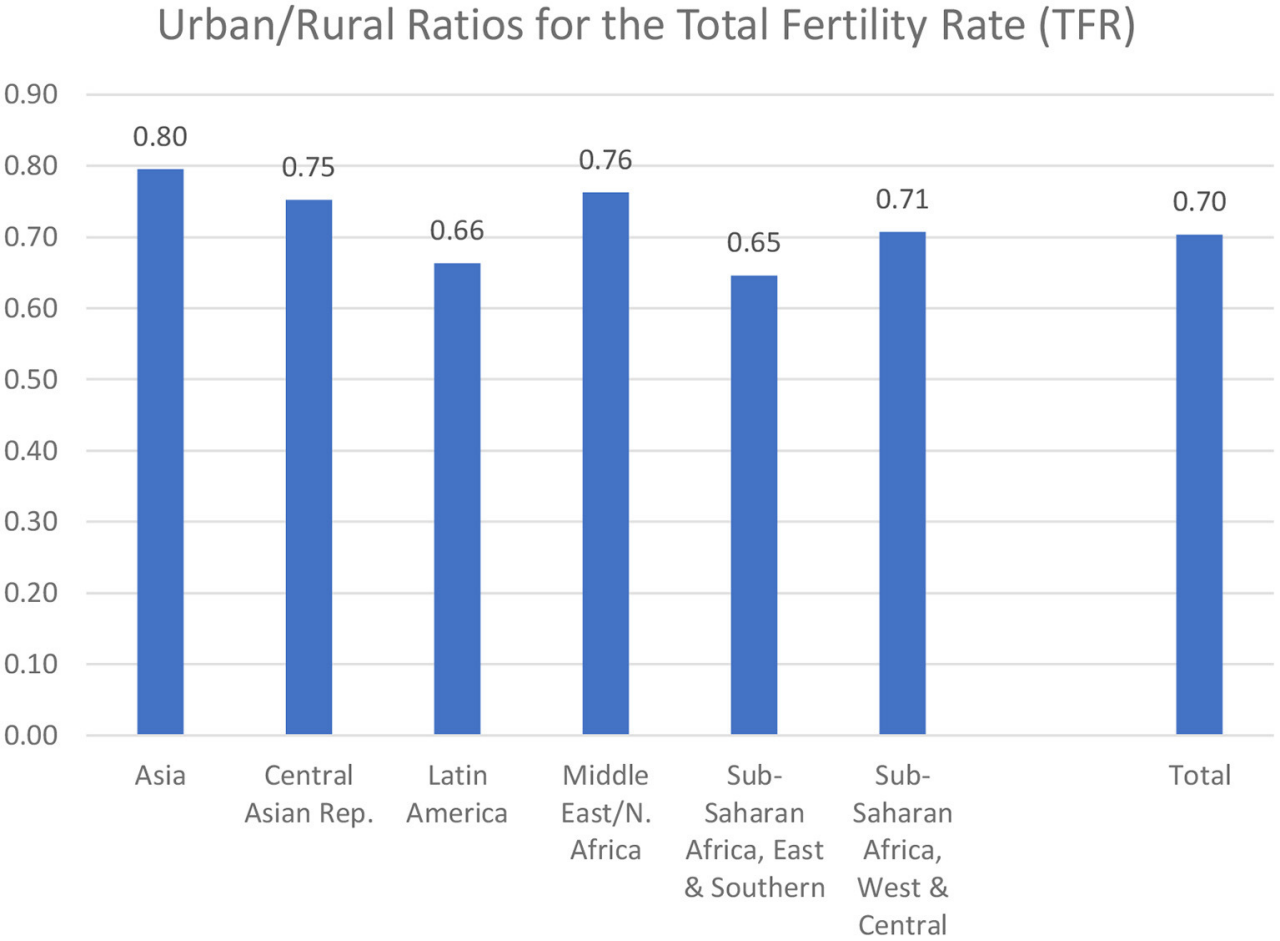
Urban/rural ratios for the Total Fertility Rate (TFR).

The regions vary considerably: in Figure 7 the difference is least in Asia, where urban fertility is 80% of rural fertility, and greatest in the East and Southern parts of sub-Saharan Africa and in Latin America. The other three regions are intermediate, at 0.71 to 0.76.

It is of interest that Asia has both low fertility rates (Figure 6) and the least difference between the urban and rural rates. However, the other regions do not follow that pattern, as the ratios are irregular in their correspondence to the levels of the rates. Indeed, the balance between the fertility behavior in the urban and rural environments is subject to somewhat different sets of determinants across the regions.

**Figure 8:**
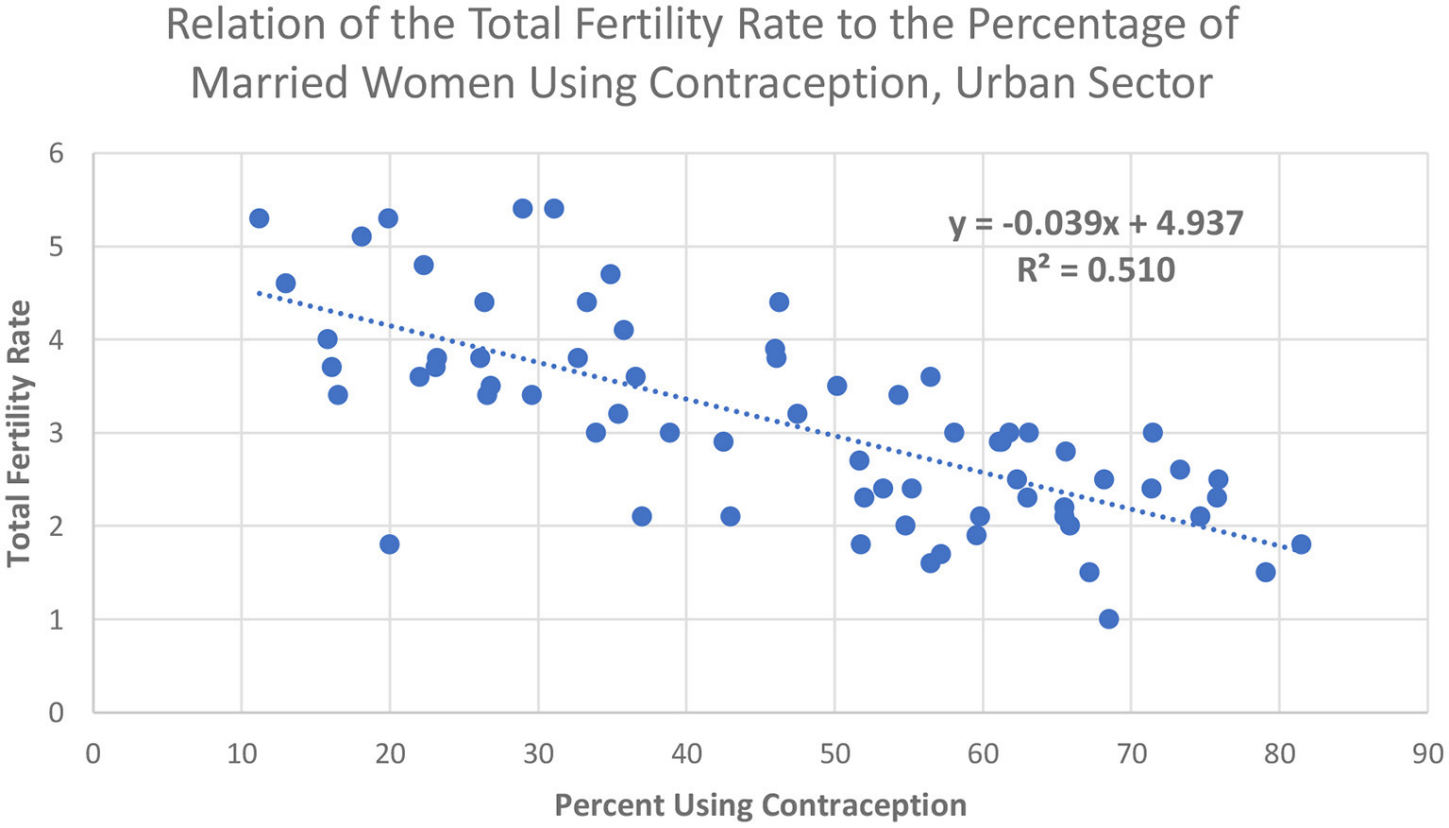
Relation of the Total Fertility Rate to the percentage of married women using contraception, urban sector.

Contraceptive use is one of those determinants. Where contraceptive use is greater, the TFR is lower (Figure 8). Most of the countries lie within one child of the trendline. The same pattern exists by an alternative measure, the general fertility rate, i.e., the number of births per year per 1,000 women (not shown). As in entire countries, behavior in the cities shows the dependence of fertility outcomes on contraceptive use. This, in turn, leads to the question of access to contraceptive methods.

### Unmarried Sexually Active Women

A group of particular interest is sexually active unmarried women. Most are young, living in the cities, exposed to unplanned pregnancies, and unsafe abortions. Many are poor and preyed upon by older men. The contraceptive methods available to them, and the ones they adopt, are limited and vary markedly across countries. However, in some countries, higher proportions of these women use contraception than do their married counterparts of the same age, partly because many young married women are seeking their first births. Measurement issues are discussed by Fabic and Jadhay (7).

Information concerning this group is available from the DHS series for 55 countries with surveys from 2000 onward. Information for the urban sector alone is not available, but on average, 62% of these women live in the cities and so it seems worthwhile to include the available information in this study since the broad patterns should apply to the urban sector. The following therefore pertains to whole countries and is only for the especially concerned age groups 15–19, 20–24, and 25–29. Appendix 3 gives the total percentage using each method across the age groups, for the 64 countries with information.

About half of the sexually active, unmarried women use some contraceptive method; nearly all of this is for modern methods (47.8% compared to 49.9%). Table 1 gives the mean values for the percentage using any method and any modern method, showing the substantial regional differences (there are only two countries with information in the Central Asia Republics, but all five are known to heavily favor the IUD).

**Table 1:** Contraceptive use by unmarried sexually active women, by all methods and modern methods, and region.

	**Any method**	**Modern methods**
Asia	30.8	29.8
Central Asia Rep.	57.8	60.3
Latin America	65.3	59.4
Sub-Saharan Africa, E & S	53.5	45.2
Sub-Saharan Africa, W & C	41.1	40.4
Total	49.9	47.8

**Figure 9:**
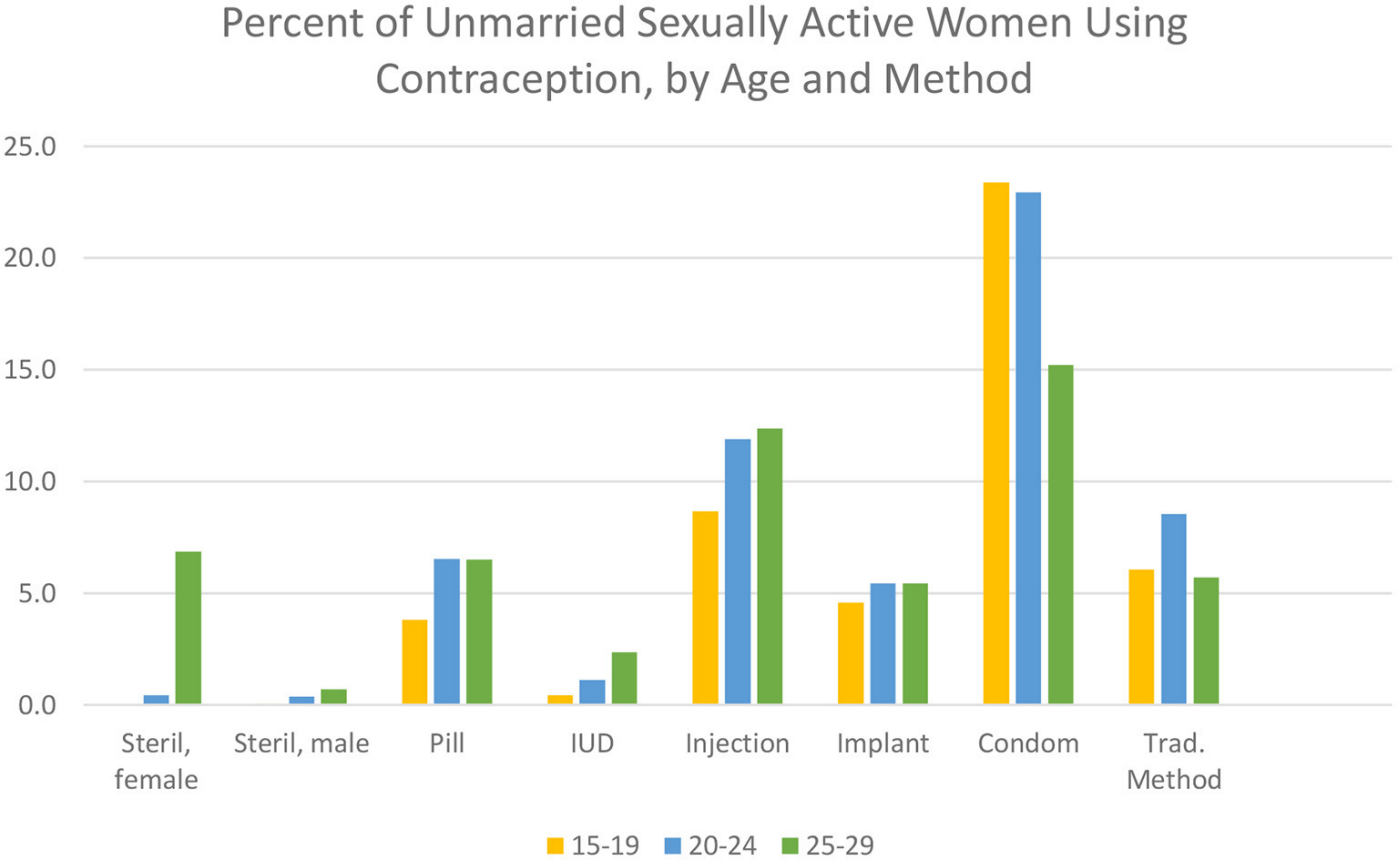
Percent of unmarried sexually active women using contraception, by age and method.

As Figure 9 shows, there are decided differences as to the preferred method, and these vary interestingly by age. The condom is easily the favorite, followed by the injectable. The pill, implant, and traditional methods are equally popular. However, the long-term methods of the IUD and sterilization are little used, although both increase in the 25–29 age group. Only the condom shows a clear decline by age.

In summary, an average of about half of unmarried, sexually active women use some method of contraception but with wide variations in the percentage using and the particular method chosen. Every method except male sterilization finds some users, and each method has its adherents. Overall, most of the use is of the resupply methods, a reasonable outcome given the young age range and the unmarried status.

Regional differences are quite marked. Appendix 3 shows the percentage of unmarried, sexually active women using each contraceptive method, by country and region. The pill is outstanding only in some Latin American countries. Notably, the injectable has won a prominent place there, but not as impressively as in most of the countries in the east and southern regions of sub-Saharan Africa. The implant is important only in selected countries there and in the west and central regions. The condom, as noted, is the most used method, true in every region except Asia. Finally, the IUD is little used except in Kazakhstan. Female sterilization is prominent only in Latin America; it is negligible elsewhere, with the remarkable exception of India, where 48% use it.

These patterns of contraceptive use among sexually active, unmarried women reflect the broader preferences of other women, but only partially. In particular, the condom is far less popular among other women.

### Family Planning Programs to Extend Contraceptive Use

Large-scale programs offering contraceptive information and services exist in most of the developing countries, often as part of maternal and child health services. These programs vary in their strength of effort, as measured by 30 ratings over the past several decades (8, 9). The ratings are summarized under four components, for policies, services, evaluation, and access to methods, and are available for 90 countries. The study is not specific to the urban population, and the data pertain to entire populations. However, it serves as a useful proxy since most of the programs serve both the urban and rural sectors, and the ratings provide at least a broad picture of differentials across regions and over time. Appendix 4 provides the latest (2014) ratings for the four components in the 90 countries studied.

The total rating for the strength of program efforts (average of the 30 indicators) has greatly improved over the four decades since the first measurement in 1972 and repeated rounds about every 5 years (probably the longest series of any indicator for family planning ever done). The increase was from a country average of 20 (of a maximum of 100) in 1972 to 58 in 2014, a near tripling. However, 58 leaves a considerable margin for improvement, and there was some leveling off after 2004.

The national programs attempt primarily to encourage the use of the “modern” methods (IUD, pill, sterilization, injectable, implant, and condom), with varying emphases among countries. These are generally advocated in preference to the “traditional” methods of rhythm and withdrawal, which require no supplies or clinical involvement but have higher failure rates. Across countries, the use of modern methods correlates at 0.50 with the total rating of effort and much higher with the rating for access to methods, at 0.75 (“*r*” values).

Differentials by the four program components and by region are quite marked (Table 2). By the total score, programs in Asia and the Central Asian Republics have scored the highest, well above the other regions. The lowest scores are for sub-Saharan Africa in the west and central subregions and in the “MENA” countries for the Middle East and North Africa. Sub-Saharan countries in the eastern and southern subregions rank near to those in Latin America. Overall (total row), policy positions score the highest, and actual services score the lowest. Evaluation activities rank second, with access close.

**Table 2:** Strength of national family planning programs.

	**Total score**	**Policies**	**Services**	**Evaluation**	**Access**
Asia	55.7	59.0	53.4	55.8	56.3
Central Asia Rep.	57.5	56.2	53.5	58.9	65.6
Middle East/N. Africa	45.4	46.3	41.5	50.0	49.7
Latin America	49.5	53.3	45.1	51.0	52.8
Sub-Saharan Africa, E & S	50.9	55.7	48.0	52.9	50.0
Sub-Saharan Africa, W & C	45.4	48.8	43.1	49.7	43.8
Total	49.9	53.0	46.8	52.3	51.4

The contributions of the programs are reflected in the source of supply or service as reported by survey respondents. As a country average, 62% of respondents report that their most recent contraceptive supply or information came from a public source, including government hospitals and health centers. Without the involvement of the national family planning programs, contraceptive use would be less available for large segments of the population. This leads to the ratings for access to the various methods.

#### Access to Methods

A critical test of the national policies favoring family planning is the proportion of the population with access to specific contraceptive methods. The access component of the ratings above is the average rating across the methods, each of which can theoretically range from 0 to 100% but in fact the ranges are from about 30% to about 75%, with large variations by region. As noted above, the correlation is as high as 0.75 between the use of modern methods and the access ratings.

A breakdown (Table 3) shows a great diversity of access by both the method and the region, from very low ratings for male sterilization in all regions and low ratings for safe abortion except in the Central Asian Republics. The ratings are evenly high values for the condom and pill. The implant scores especially high in sub-Saharan Africa but not elsewhere, so its total rating is low. Indeed, most of the methods vary substantially by region: the IUD is highly favored in the Central Asian Republics and considerably so in the Middle East/North African countries, where the injectable, however, shows the lowest access. Overall, the West/Central countries in sub-Saharan rate the lowest by a substantial margin.

**Table 3:** Percent with access to each method.

	**Steril, female**	**Steril, male**	**IUD**	**Implant**	**Pill**	**Injection**	**Condom**	**Safe abortion**
Asia	48.9	38.3	57.4	41.1	75.4	61.4	78.1	34.4
Central Asia Rep.	51.2	26.1	84.1	31.5	79.9	63.7	83.9	70.4
Middle East/N. Africa	35.5	15.3	66.6	25.0	74.3	50.8	74.8	30.8
Latin America	49.8	27.3	56.8	28.3	75.5	69.7	78.6	11.9
Sub-Saharan Africa, E & S	35.9	22.8	44.0	49.0	76.4	72.1	82.5	16.5
Sub-Saharan Africa, W & C	20.6	11.0	46.6	48.7	70.0	62.8	80.9	14.8
Total	38.7	23.2	55.6	38.5	74.7	63.6	79.4	24.4

The upshot from these uneven patterns is that countries have a far way to go to ensure ready access to a variety of methods, augmenting resupply (short-term) methods with long-term (clinical) methods such as the IUD, implant, and sterilization.

#### Access Ratings by Country

**Figure 10:**
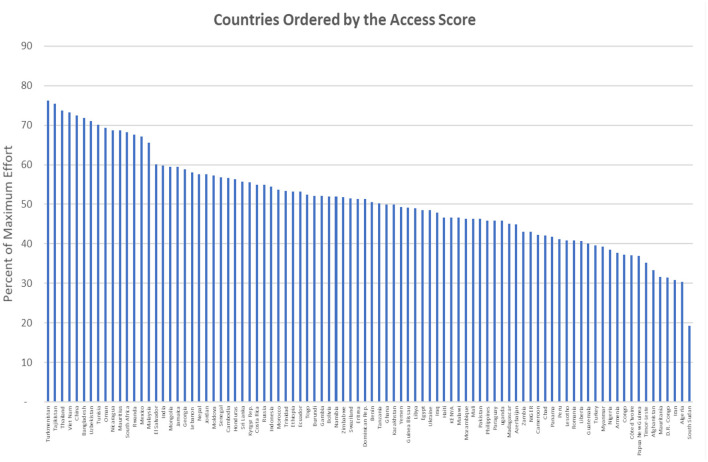
Countries ordered by the access score.

The diversity by region is also common at the country level. Figure 10 shows the proportion of the population in each country based on the same access score as in Table 2. Higher access ratings are found in such Asian countries as China, Bangladesh, and Vietnam, as well as in the Central Asian Republics of Tajikistan, Turkmenistan, and Uzbekistan. However, they are quite low (below 40%) in Afghanistan, Nigeria, the D.R. Congo, and South Sudan, among others.

The overall distribution of the access scores across the countries runs from the mid-70's to the mid-40's around a median of 51.0% (mean 51.2%), where 100% indicates that the entire population has ready access to the methods. (The interquartile range is from 42.8 to 57.5%, just seven points above and below the median.). The total range runs from a low of only 19.3% to 76.2%. Indeed, there is much room for improvement, where even the best-scoring countries fall short, about 25% below full access.

## Discussion

Contraceptive use is higher in the cities, and the method mix is somewhat different from that in rural areas. Fertility rates are also lower, and they correlate with the extent of contraceptive use. Additional factors are smaller desired family sizes, later marriage, and reliance upon abortion. Separate data on abortions in the cities are lacking, but country estimates show them to be important and highly variable (10). Among sexually active unmarried women, about half use some method, and the variety of method preferences is remarkable.

All of these findings are highly variable by region and by countries within each region, as shown, for example, by the leaning toward the IUD in the Middle East/North Africa and the Central Asia Republics, toward sterilization in the Indian subcontinent and the implant and injectable in sub-Saharan Africa.

The urban environment offers logistic advantages for contraceptive supply and more clinical facilities for methods, as well as a more active private sector, more mass media activity, and easier educational approaches. National family planning programs have gained strength over the decades, but ratings show them to be, on average, about half of full strength. This is also true of the access component of the effort, regarding the availability of the methods to the general population. All this leaves substantial gaps between current performance and full access to contraceptive protection.

For planning purposes by local and international agencies, the watchword, therefore, is an examination of the particular profile of use and method availability in each country. The main patterns of method preferences will not change appreciably, although the advent of the injectable and implant in sub-Saharan Africa shows the potential of introducing new technologies and making them affordable and available.

The question of equity for contraception across lines of social status is not directly discussed in this report; however, the differentials by wealth quintiles show less use by the poor and a selective method mix, and the poor are likely to suffer from disadvantages in access to a full choice of contraceptive options. A broader look at equity for both contraception and 18 reproductive health indicators found a welcome move toward diminishing gaps between the poor and the rich (11). The narrowing of the gaps was primarily due to faster improvements among the poor than among the rich.

The effects of the ongoing Covid virus pandemic deserve attention. To some extent, it disturbs the relationships and findings discussed above by the morbidity and mortality that it currently causes, just as historically the HIV epidemic did to reproductive behavior. In both cases, the effects were compounded because the medical staff who were required to fight the disease were themselves weakened. And in both cases, the poorest and most disadvantaged members of the public were the most affected, worsening the equity imbalances already present.

## Data Availability Statement

Publicly available datasets were analyzed in this study. This data can be found at: https://www.statcompiler.com/en/.

## Author Contributions

JR designed the analysis and wrote the article.

## Conflict of Interest

The author declares that the research was conducted in the absence of any commercial or financial relationships that could be construed as a potential conflict of interest.

## Publisher's Note

All claims expressed in this article are solely those of the authors and do not necessarily represent those of their affiliated organizations, or those of the publisher, the editors and the reviewers. Any product that may be evaluated in this article, or claim that may be made by its manufacturer, is not guaranteed or endorsed by the publisher.

## Footnotes

^1^The UN Population Division provides estimates and projections of both urban and rural population numbers for every country and region, 1950–2050. Online at population.un.org/wup.

^2^In order of urban population size in 2020: Brazil, Indonesia, Mexico, Pakistan, Bangladesh, Turkey, Iran, Philippines, Egypt, Republic of Korea.

^3^For details, see http://ghdx.healthdata.org/series/world-fertility-survey-wfs.

^4^For a compilation of all national surveys from these and other sources, refer to https://www.un.org/development/desa/pd/data/world-contraceptive-use.

^5^Wealth for the respondent is according to that of her household, which is judged by the ownership of selected assets such as televisions and bicycles; materials used for housing construction; and types of water access and sanitation facilitation facilities. Details at https://dhsprogram.com/topics/wealth-index/Wealth-Index-Construction.cfm.
